# Lactoferrin from Milk: Nutraceutical and Pharmacological Properties

**DOI:** 10.3390/ph9040061

**Published:** 2016-09-27

**Authors:** Francesco Giansanti, Gloria Panella, Loris Leboffe, Giovanni Antonini

**Affiliations:** 1 Department of Health, Life and Environmental Sciences, University of L’Aquila, L’Aquila I-67100, Italy; gloria.panella86@gmail.com; 2 Interuniversity Consortium on Biostructures and Biosystems INBB, Rome I-00136, Italy; giovanni.antonini@uniroma3.it; 3 Department of Sciences, Roma Tre University, Rome I-00146, Italy; loris.leboffe@uniroma3.it

**Keywords:** lactoferrin, transferrins, nutraceutical, iron

## Abstract

Lactoferrin is an iron-binding protein present in large quantities in colostrum and in breast milk, in external secretions and in polymorphonuclear leukocytes. Lactoferrin’s main function is non-immune protection. Among several protective activities shown by lactoferrin, those displayed by orally administered lactoferrin are: (i) antimicrobial activity, which has been presumed due to iron deprivation, but more recently attributed also to a specific interaction with the bacterial cell wall and extended to viruses and parasites; (ii) immunomodulatory activity, with a direct effect on the development of the immune system in the newborn, together with a specific antinflammatory effects; (iii) a more recently discovered anticancer activity. It is worth noting that most of the protective activities of lactoferrin have been found, sometimes to a greater extent, also in peptides derived from limited proteolysis of lactoferrin that could be generated after lactoferrin ingestion. Lactoferrin could therefore be considered an ideal nutraceutic product because of its relatively cheap production from bovine milk and of its widely recognized tolerance after ingestion, along with its well demonstrated protective activities. The most important protective activities shown by orally administered bovine lactoferrin are reviewed in this article.

## 1. Introducing Lactoferrin

Lactoferrin (Lf, formerly known as lactotransferrin) is an iron-binding glycoprotein, belonging to the transferrin protein family, together with serum transferrin (sTf), ovotransferrin (Otrf), melanotransferrin and the inhibitor of carbonic anhydrase. Lf was first isolated by Sorensen and Sorensen from bovine milk in 1939 and after two decades it was determined to be the main iron- binding protein in human milk [1,2]. Lf is produced and released by mucosal epithelial cells and neutrophils in various mammalian species, including humans, bovines, cows, goats, horses, dogs and several rodents. More recently [3] González-Chávez et al. showed that Lf is also expressed in fishes. 

Lf has been shown to be involved in several physiological and protective functions, including regulation of iron absorption in the bowel; antioxidant, anticancer, antinflammatory and antimicrobial activities, which are the most widely studied function to date [4,5,6,7,8,9,10,11,12]. Interestingly, a great number of Lf activities are present also in ovotransferrin, an avian homologue of mammalian Lf, indicating that they have been conserved during evolution [13,14]. Beside mammalian milk and colostrum, where Lf, present at a concentration of 7 g/L, is the second most abundant protein after caseins [15,16], Lf is primarily found in mucosal secretions; in particular it is present in tears, saliva, vaginal fluids, semen [17], nasal and bronchial secretions, bile, gastrointestinal fluids, and urine [18]. It is also found in considerable amounts in secondary neutrophil granules (15 µg/10^6^ neutrophils) [19], where it plays a significant physiological role and it can also be found in bodily fluids such as blood plasma and amniotic fluid.

**Figure 1:**
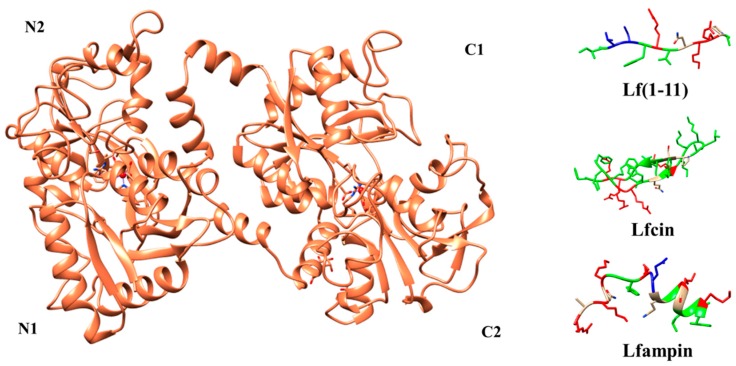
Three-dimensional structures of holo-bLf (PDB ID:1BLF), Lf(1–11), Lfcin (PDB ID: 1LFC), and Lfampin (PDB ID: 2MD1). N1, N2, C1, and C2 indicate the subdomains of each lobe. The iron atom is shown as a red sphere, while the interacting amino acid residues of Lf are highlighted. Lf(1–11) is shown with the conformation it has in the intact protein (i.e., bovine lactoferrin) and the missing residues have been added with MODELLER-9.15 [24]. The colors of peptides indicate aminoacid properties: Green: hydrophobic; Blue: negatively charged; Red: positively charged; White: polar. For details, see text. The pictures were drawn by UCSF-Chimera package [25].

Lf is an 80 kDa glycosylated protein of about 700 aminoacids (711 aa for human Lf (hLf) and 689 aa for bovine Lf (bLf)) with high homology among species. Its three dimensional structure consists of a single polypeptide chain folded into two symmetrical lobes (N and C lobes), which are highly homologous with one another (33%–41% homology) (Figure 1) [10,20,21,22,23].

A hinge region containing parts of an α-helix connects the two lobes. In hLf, the polypeptide chain includes amino acids 1–332 for the N lobe and 344–703 for the C lobe and it is made up of α-helix and β-sheet structures. Each lobe can be further divided into two subdomains (N1 and N2 in the N-lobe and C1 and C2 in the C-lobe) forming two clefts for each lobe, inside of which the iron is bound [23]. In hLf, the subdomain N1 contains the residues 1–90 and 251–333, while N2 contains the residues 91–250) [10,20,23]. Each iron ion is bound to the protein together with two carbonate ions (CO_3_^2−^) whose protonation leads to iron binding weaknesses [20,26]. However, the binding site is not strictly restricted to iron, since Lf is able to bind other metals such as Cu^2+^, Zn^2+^ and Mn^2+^ ions [27,28,29]. In biological fluids, Lf can be found either without bound iron (apo-lactoferrin) or in iron-bound form (holo-lactoferrin) [30].

A large number of studies have demonstrated its tolerance in humans, and bLf has been approved by FDA (US) and European Food Safety Authority as a dietary supplement in food products [31,32]. However, as Lf is susceptible to peptic digestion in the stomach, it cannot easily access to the digestive tract. To overcome this problem and to improve the therapeutic potential of Lf, the use of microparticles or liposomes has been tested [33]. Liposomalization increased the Lf action by the improvement of stability against gastric degradation and facilitated the interaction with the intestinal membrane and with Lf-specific receptors [33,34].

## 2. Lactoferrin’s Antimicrobial Activity

Lf’s protection against microbial infection was the first activity discovered and it is currently the most widely studied function to date [4,5,6,7,8,9,10,11]. There is a huge amount of literature data describing the in vitro and in vivo efficacy and animal-model benefits of Lf. The antimicrobial activity of Lf is due to two different mechanisms. Its primary role is to sequester free iron, thus removing an essential substrate required for bacterial growth and exerting a bacteriostatic effect. The other mechanism involves a direct interaction of the Lf with the infectious agent. Lf binds to the lipopolysaccharide of bacterial walls, and may also damage bacteria via formation of peroxides catalyzed by Lf-bound iron (III) ions, affecting membrane permeability and resulting in bacterial cell lysis [19,35,36]. Since the pioneristic work of Bullen and co-workers [37] on the protective effect of Lf towards *E. coli* 0111 infection in newborn guinea-pigs, there are several experimental observations that oral administration of Lf reduces bacterial and fungal infections mainly in the gastro-intestinal tract (see, for example, [9,38,39,40,41,42,43,44]).

On the other hand, Lf promotes the growth of bacteria with low iron requirements such as *Lactobacillus* and *Bifidobacteria*, which are generally believed to be beneficial to the host [45]. Orally administered recombinant human Lf (rhLf) was indeed demonstrated to be able to modulate the intestinal flora in piglets [46]. Recently oral administration of bLf has had a significant increase of interest because of the demonstration of its probiotic activity both in adults and in infants, including prematurely born [47,48,49].

In addition to this well-known antibacterial function, Lf displays a potent antiviral activity of against both enveloped and naked viruses, like *Cytomegalovirus* (CMV) [50,51], *Herpes simplex virus* (HSV) [9,52,53,54], *Human immunodeficiency virus* (HIV) [55,56,57], *Human hepatitis C* (HCV) and *human hepatitis B* (HBV) viruses [58,59]. Therefore, the effects of Lf oral administration have been studied in several bacterial and viral infections in animals and humans and the beneficial effects of orally administered Lf have recently been found in viral infections including the common cold, influenza, and viral gastroenteritis [60,61,62,63].

An antiparasitic activity of Lf has been also demonstrated. This antiparasitic activity appears to involve interference with iron acquisition in some parasites, e.g., *Pneumocystis carinii* [64,65], on the other hand, Lf appears to act as a specific iron donor in other parasites such as *Tritrichomonas foetus* [66]. Furthermore, Lf is able to inhibit the growth of *Plasmodium berghei*, binding directly to the plasmodial CS protein [67]. Moreover, Lf has in vitro activity towards human pathogenic fungi, such as *Candida albicans* and several other *Candida species* [11,68].

## 3. Nutraceutical and Immunomodulation Protective Effects

Over the past decade, Lf has been reported to affect various immunological functions playing an important role in host defence against infection and excessive inflammation, appearing as a key element in the mammalian immune system [69]. Indeed, Lf appears to be able to both up- and down-regulate the endogenous inflammatory response, possessing pro- and anti-inflammatory properties [70]. The cellular and molecular mechanisms responsible for the immunomodulatory effects of Lf, are not fully elucidated, and in vitro and in vivo studies suggest the existence of multiple mechanisms [71,72,73,74]. Lf acts on B-cells, which are well-known antigen presenters, to allow their subsequent interaction with T cells, which promotes elevation of the antibody response [75] by promoting the maturation of T-cell precursors into competent helper cells and by the differentiation of immature B-cells into efficient antigen presenting cells [72]. Both bovine and human Lf are able to bind surface receptors on the human T-cell line (Jurkat cell line) [76] and the expression of Lf receptors has been recently reported in all T-cell subsets [77]. At the cellular level, Lf modulates the migration, maturation and function of immune cells [78]. There are also suggestions that Lf can play a role in the initiation of T-cell activation through the modulation of dendritic cell function [79]. Several studies (mostly on animals but also on humans) have focused on the immunomodulatory effects of orally administered Lf:

- Mulder et al. [80] found that oral administration of bLf to healthy human males increased the activation of total T-cell, helper T-cell and cytotoxic T-cell, increasing also hydrophilic antioxidant capacity (reviewed by Mayeur et al. in 2016 [81]).
- Saraiva et al. [82] fed piglets with 3.6 g/L of bLF and showed higher IgG concentrations in serum and more IL-10, an immunomodulatory cytokine potentially limiting inflammation, was secreted by spleen cells into the culture media;
- Liu et al. [83] administering orally bLf to piglets, and found an increase of the blood NK cell populations and NK Lf receptor expression without affecting NK cell cytotoxicity, suggesting that Lf could help protect the organism of infants from infections;
- Cooper et al. [84] fed young pigs with transgenic cows’ milk containing rhLf. They showed favorable changes in systemic health in rhLf-milk fed pigs that had beneficial changes in circulating leukocyte populations with a decrease in neutrophils and increase in lymphocytes which is an indicator of decreased systemic inflammation. Moreover, favorable changes in intestinal villi architecture were also observed both in the duodenum and in the ileum of rhLf-milk fed pigs;
- Yang et al. [85] showed that the percentage of piglets with symptoms of diarrhea during the first 38 days of life was decreased, if compared with the control group, from 54% to 15% by orally administered Lf at a dose level of 155 and 285 mg/kg/day, respectively. A significant delay in the onset of diarrhea by at least 1 week in the higher Lf dose group and 4 days in the lower Lf dose group, compared with the control group of piglets, was also observed;
- Wu et al. [86] investigated the effect of enteral bLf supplementation on intestinal adaptation and barrier function in a rat model of short bowel syndrome (SBS) and they demonstrated a protective effect of Lf due to small-bowel luminal sIgA and TJ protein expression upregulation together with reduced intestinal permeability, supporting intestinal barrier integrity and providing better protection against bacterial infections;
- Arciniega-Martınez et al. [87] analyzed the effects of bLf orally administered to healthy male BALB/c mice. They found that antibodies, antibody-secreting cells, and B and T responses in both Peyer’s patches and in lamina propria were higher in bLf-treated than bLf-untreated mice, suggesting a potential application of bLf as a nutraceutical to control inflammation in the distal small intestine;
- Kawashima et al. [88] demonstrated the protective effect of Lf towards “Dry Eye Syndrome” caused by age-induced decrease in lacrimal gland secretory function. They attributed this activity to Lf anti-inflammatory properties since oral administration to aged mice of Lf alone or in combination with other antioxidants resulted in decreasing inflammatory cell infiltration in eyes. On the other hand [89] they demonstrated also that Lactoferrin administration decreases MCP-1 and TNF-α expression levels and markers for oxidative damage while increases the volume of tear secretion. Moreover, a combined dietary supplement containing fish oil, lactoferrin, zinc, vitamin C, lutein, vitamin E, γ-aminobutanoic acid, and *Enterococcus faecium* WB2000 improves the symptoms of dry eye syndrome with no side effects [90].

However, despite the abundant evidence that orally administered Lf indeed has direct and indirect effects against inflammation, up to now it was not possible to understand the mechanisms of action of Lf that appear to be controversial in a lot of cases. In addition, several approaches have been used to study the way in which lactoferrin mediates neonatal immune development. One of these was to create transgenic animals that overexpress Lf in milk, which has been done in mice, goats, and cows as reviewed by Cooper et al. [91]. 

## 4. Anticancer Activity of Orally Administered Lactoferrin

The process of carcinogenesis includes the initial occurrence of genetic alterations which can lead to the inactivation of tumor suppressor genes and further accumulation of genetic alterations during tumor progression [91]. Silencing or downregulation of the lactoferrin gene in cells is usually related to the occurrence of certain diseases, especially carcinomas [92,93]. 

Table 1 summarizes the identified anticancer activities of Lf and its derivative peptides, among which the lactoferricin (Lfcin) (see paragraph *Lactoferrin peptides*). Lf, and subsequently its peptides, is one of the most studied nutraceutical proteins, showing considerable potential for preventing the different stages of cancer, including initiation, promotion, and progression. Usually, alterations of the lactoferrin gene in cells are associated with an increased incidence of cancer. Several studies suggest that exogenous treatment with Lf and its derivatives can efficiently inhibit the growth of tumors and reduces susceptibility to cancer [115,116]. The effect of apo- and holo-Lf in inhibiting tumor development and reducing growth and metastasis of solid tumors was also observed by Kanwar et al. and Tsuda et al. [117,118,119]. 

Actually, Lf and its derivatives can be an effective anti-cancer treatment if combined with other therapeutic agents or if encapsulated in carriers after appropriate modifications. It is interesting to note that some studies have already begun to use these approaches to increase the cytotoxic effects of Lf and its derivatives [120,121,122,123,124]. Oral Lf accelerated reconstitution of humoral and cellular immune responses during chemotherapy- induced immunosuppression in mice, suggesting it could be employed to overcome tamoxifen-induced immune suppression [116,117] and indeed, Kanwar and coworwers showed that the oral administration of holo-bLf could improve the chemotherapeutic effects of tamoxifen in the treatment of ER-negative breast cancers [117]. Orally administered holo-bLf improves the chemotherapeutic effects of tamoxifen in the treatment of basal-like breast cancer in mice inducing significantly higher cancer cell death (apoptosis) and cytotoxicity in a dose-dependent manner in cancer cells than the normal intestinal cells [125]. 

As combination therapy becomes increasingly popular, it is likely that Lf will continue to be studied for its potential value as a primary or adjuvant agent in the treatment of cancer [12]. Clinical trials involving the use of this protein in cancer therapy are ongoing and the relatively low cytotoxicity of Lf and its derivatives as compared with known anticancer drugs, along with the lack of data about the mechanisms of action, is likely to encourage the clinical use of Lf in cancer treatment. 

## 5. Other Lactoferrin Activities 

In addition, there is a potential use of Lf for the improvement of bone health. Dietary Lf supplementation preserved bone mass and microarchitecture in ovariectomized rats, improving both bone mineral density and bone strength [126]. More importantly, in 38 healthy postmenopausal women, milk ribonuclease-enriched lactoferrin induces positive effects on bone turnover markers [127]. Lf appeared therefore to be a promising candidate for the development of an anabolic therapeutic factor for osteoporosis [128], even though no more recent information is present in scientific literature.

Maternal iron-bLf supplementation stimulates human fetal growth in normal pregnancy [129]. In addition, iron-bLf administration is able to decrease the onset of dexamethasone (DEX) induced IntraUterine Growth Restriction (IUGR) that alters levels of brain metabolites (γ-aminobutyric acid, glutamate, *N*-acetylaspartate, and *N*-acetylaspartylglutamate) and transcripts (brain-derived neurotrophic factor (BDNF), divalent metal transporter 1 (DMT-1), and glutamate receptors), leading to defective hippocampal development and later cognitive impairment [130].

## 6. Lactoferrin Peptides

An increasing number of functions are associated with the peptides of this protein. These peptides are produced by the action of proteolytic enzymes, and could be produced in the intestinal lumen after Lf oral ingestion. In particular, three peptides have been especially studied. They originate from the N-lobe of Lf, and have an antimicrobial activity by their hydrophobicity, cationic charge, and helical conformation, which render these peptides amphiphilic molecules.

These three peptides are Lf(1–11), Lfcin and lactoferrampin (Lfampin). In detail, Lf(1–11) is the oligopeptide that includes the first eleven aminoacidic residues of the Lf. The presence of the hydrophobic and hydrophilic residues lends to it a highly cationic nature. It has been demonstrated that Lf(1–11) interacts with the membrane of several bacteria.

Lfcin is an amphipathic, cationic peptide with anti-microbial and anti-cancer properties. Indeed it is the most studied anti-microbial peptide derived from milk proteins. It can be generated by the pepsin-mediated digestion of Lf (aminoacid residues 17–41). The peptide has an abundance of basic aminoacids including Lys and Arg, as well as hydrophobic residues like tryptophan and phenylalanine. Lfcin has several biological activities including antiviral, antibacterial, antifungal and anti-inflammatory activities.

Lfampin comprises residues 268–284 in the N1 domain of Lf, and was found to be located in closed proximity to Lfcin. Lfampin exhibits broad antimicrobial action against several Gram-positive and Gram-negative bacteria, yeast and parasites. 

Lf- derived peptides showed often a considerably higher antimicrobial activity than the native protein with broad antibacterial spectrum and low minimum concentration [131]. However, their activities are not limited to the antimicrobial properties but several protective activities have been found in Lf- derived peptides. These activities are summarized in Table 2.

## 7. Conclusions

Lf has been studied since long time for its various protective activities for which the protein has been evolved in a wide range of animals, including humans. The most beneficial activities are those related to the antimicrobial, antinflammatory and anticancer properties of the protein and of its peptides that may be possibly generated after ingestion. In addition, there is growing evidence of specific immunomodulatory activity of Lf in adults and newborns where, beyond the well-known nutritional immunity, Lf is of particular importance for the development of the immune system. These activities were shown after oral administration of bovine and human Lf and, together with its probiotic effect and the lack of any specific toxicity, allow Lf to be an ideal nutraceutical example. 

## Figures and Tables

**Table 1:** Anticancer activities of lactoferrin and lactoferricin.

Cancer Type	Mechanism of Anticancer Action	References
Breast	hLf causes arrest in the G0/G1 phase, induction of cell apoptosis and regulation of the expression of Bcl-2, Bax and activation of caspase 3.	[94]
Cervix	hLf inhibits cervical cancer due to elevated expression of Fas and decreased the ratio of anti- to pro-apoptotic molecule Bcl-2/Bax.	[95]
Colon	Lfcin causes arrest in the at S phase through downregulation of cyclin E1 in CaCO_2_ cells.	[96]
	hLF increases expression of TGF-β1, and holo-forms of LFs stimulate IL-18 secretion in CaCO_2_ cells.	[97]
	Lf induces caspase-1 and IL-18.	[98]
	bLf increases production of CD4+, CD8+, and IL-18	[99]
Gastric	BLfcin induces apoptosis human gastric cancer cell line AGS.	[100]
Head, neck, and oral	Lf induces suppression of AKT signaling via inhibition of 3-phosphoinositide-dependent protein kinase-1 expression and/or blocking of the K18-14-3-3 complex.	[101]
	bLf and [Polyphenon-B (P-B)] P-B was more effective in inhibiting hamster buccal pouch (HBP) carcinogenesis by inhibiting oxidative DNA damage, carcinogen activation, cell proliferation, invasion, and angiogenesis.	[102]
	Lf inhibits tumor through direct cellular inhibition and immunomodulation.	[103]
	Lf causes cell cycle arrest through downregulation of cyclin-dependent kinases and upregulation of p27 protein expression in head and neck cancer cell lines.	[104]
	Lf derivated peptides induce apoptosis via JNK/SAPK activation in squamous cell carcinoma cell line SAS.	[105]
Leukemia	LfcinB6 (RRWQWR) induces citoxicity via caspase-mediated and cathepsin B-mediated mechanism in T-leukemia cells.	[106]
	Lfcin kills T-leukemia cells by triggering the mitochondrial pathway of apoptosis and through the generation of reactive oxygen species.	[107]
	LF11-322 (PFWRIRIRR-NH2), peptide fragment derived from human lactoferricin, induces necrosis in leukemia cells (MEL and HL-60 leukemia cells).	[108]
	Lf increases CDK6 and hyper-phosphorylated retinoblastoma protein, resulting in the induction of E2F1-dependent apoptosis in Jurkat human leukemia T lymphocytes.	[109]
Lung	bLf inhibits NNK-induced mouse lung tumorigenesis, through the modification of cell proliferation and/or apoptosis.	[110]
	hLf inhibits the growth of head and neck squamous cell carcinoma via direct cellular inhibition as well as systemically via immunomodulation.	[103]
	Lf shows antiproliferative effects via hypophosphorylation of Rb on H1299 cells.	[111]
	Lfcin inhibits VEGF expression and induces apoptosis on non-small cell lung cancer H460.	[112]
NCS	Lfcin inhibits tumor growth and induces apoptosis through activation of caspases in neuroblastoma cells and in vivo).	[113]
	Lf causes growth inhibition in the NMD and FN primary cell lines and in the U87MG continuous cell line (downregulation of cyclin D1 and D4). Administration of hLf with TMZ enhanced the effect of chemotherapy both in vitro and in vivo.	[114]

**Table 2:** Biological activities of lactoferrin peptides.

Activity		Peptide	References
Antibacterial	Gram positive	Lf(1–11)	[132]
		Lfcin	[133,134,135]
		Lfampin	[134]
	Gram negative	Lf(1–11)	[136]
		Lfcin	[137,138,139]
		Lfampin	[140,141,142]
Antiviral		Lf(1–11)	[143]
		Lfcin	[54,144,145,146,147]
		Lfampin	[143]
Antifungal		Lf(1–11)	[148]
		Lfcin	[149,150]
		Lfampin	[151,152]
Antiparasitic		Lfcin	[153]
		Lfampin	[154]
Anticancer		Lfcin	[155,156,157,158]
